# 4-(4-Fluoro­phen­yl)-3-(pyridin-4-yl)-1-(2,4,6-trichloro­phen­yl)-1*H*-pyrazol-5-amine

**DOI:** 10.1107/S1600536812033569

**Published:** 2012-08-01

**Authors:** Bassam Abu Thaher, Pierre Koch, Dieter Schollmeyer, Stefan Laufer

**Affiliations:** a Faculty of Science, Chemistry Department, Islamic University of Gaza, Gaza Strip, Palestinian Territories; b Institute of Pharmacy, Department of Pharmaceutical and Medicinal Chemistry, Eberhard Karls University Tübingen, Auf der Morgenstelle 8, 72076 Tübingen, Germany; c Department of Organic Chemistry, Johannes Gutenberg-University Mainz, Duesbergweg 10-14, D-55128 Mainz, Germany

## Abstract

In the title compound, C_20_H_12_Cl_3_FN_4_, the pyrazole ring forms dihedral angles of 47.51 (9), 47.37 (9) and 74.37 (9)° with the directly attached 4-fluoro­phenyl, pyridine and 2,4,6-trichloro­phenyl rings, respectively. Only one of the two amino H atoms is involved in hydrogen bonding. The crystal packing is characterized by N—H⋯N hydrogen bonds, which result in infinite chains parallel to the *c* axis.

## Related literature

For the inhibitory activity and preparation of the title compound, see: Abu Thaher *et al.* (2012*a* ▶). For related structures, see: Abu Thaher *et al.* (2012*b* ▶,*c* ▶,*d* ▶,*e* ▶).
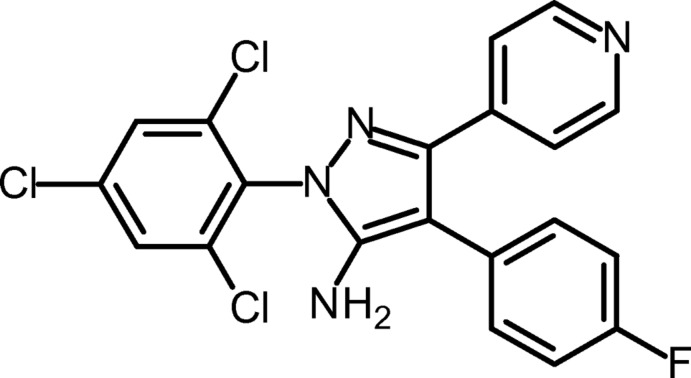


## Experimental

### 

#### Crystal data

C_20_H_12_Cl_3_FN_4_
*M* *_r_* = 433.69
Triclinic, 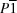

*a* = 10.2487 (5) Å
*b* = 10.4643 (5) Å
*c* = 10.5489 (5) Å
α = 109.2377 (10)°
β = 111.4008 (10)°
γ = 98.0304 (11)°
*V* = 950.03 (8) Å^3^
*Z* = 2
Mo *K*α radiation
μ = 0.51 mm^−1^
*T* = 173 K
0.33 × 0.28 × 0.07 mm

#### Data collection

Bruker SMART APEXII diffractometer
Absorption correction: multi-scan (*SADABS*; Bruker, 2006 ▶) *T*_min_ = 0.687, *T*_max_ = 0.746
21201 measured reflections
4517 independent reflections
3864 reflections with *I* > 2σ(*I*)
*R*_int_ = 0.029

#### Refinement

*R*[*F* ^2^ > 2σ(*F* ^2^)] = 0.031
*wR*(*F* ^2^) = 0.079
*S* = 1.03
4517 reflections
253 parameters
H-atom parameters constrained
Δρ_max_ = 0.33 e Å^−3^
Δρ_min_ = −0.33 e Å^−3^

### 

Data collection: *APEX2* (Bruker, 2006 ▶); cell refinement: *SAINT* (Bruker, 2006 ▶); data reduction: *SAINT*; program(s) used to solve structure: *SIR97* (Altomare *et al.*, 1999 ▶); program(s) used to refine structure: *SHELXL97* (Sheldrick, 2008 ▶); molecular graphics: *PLATON* (Spek, 2009 ▶); software used to prepare material for publication: *PLATON*.

## Supplementary Material

Crystal structure: contains datablock(s) I, global. DOI: 10.1107/S1600536812033569/bt5984sup1.cif

Structure factors: contains datablock(s) I. DOI: 10.1107/S1600536812033569/bt5984Isup2.hkl

Supplementary material file. DOI: 10.1107/S1600536812033569/bt5984Isup3.cml

Additional supplementary materials:  crystallographic information; 3D view; checkCIF report

## Figures and Tables

**Table 1:** Hydrogen-bond geometry (Å, °)

*D*—H⋯*A*	*D*—H	H⋯*A*	*D*⋯*A*	*D*—H⋯*A*
N6—H6*A*⋯N22^i^	0.90	2.17	3.0275 (17)	157

Symmetry code: (i) 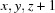.

**Fig. 1.**
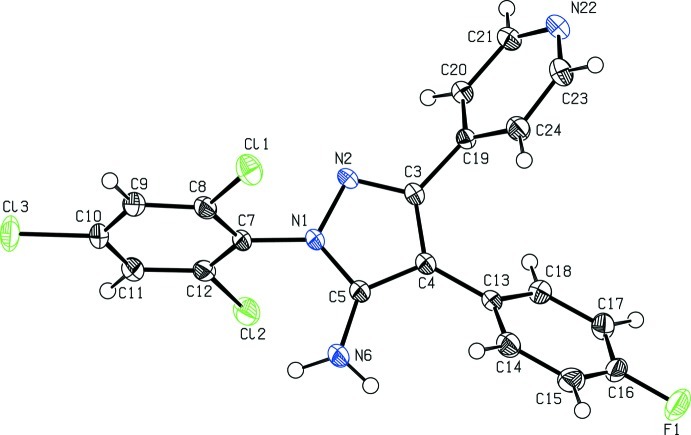
View of the title compound. Displacement ellipsoids are drawn at the 50% probability level. H atoms are depicted as circles of arbitrary size.

## supplementary crystallographic information

### Comment

The regioisomeric switch from 3-(4-fluorophenyl)-4-(pyridin-4-yl)-1-(aryl)-1*H*-pyrazol-5-amine to 4-(4-fluorophenyl)-3-(pyridin-4-yl)-1-(aryl)-1*H*-pyrazol-5-amine changes the inhibitory profile from p38α MAP kinase to kinases relevant in cancer (Abu Thaher *et al.* 2012*a*). Recently, we reported similar crystal structures (Abu Thaher *et al.* 2012*b*,*c*,*d*,*e*). In the crystal structure of the title compound (Fig. 1), the pyrazole ring forms dihedral angels of 47.51 (9)°, 47.37 (9)°, 74.37 (9)° with the 4-fluorophenyl, pyridine and 2,4,6-trichlorophenyl rings, respectively. The 4-fluorophenyl ring encloses dihedral angels of 64.25 (8)° and 66.11 (8)° toward the pyridine and 2,4,6-trichlorophenyl rings, respectively. The pyridine ring is orientated at a dihedral angle of 78.99 (8)° toward the 2,4,6-trichlorophenyl ring. The crystal packing shows that the amino function acts as a hydrogen bond donor of an intermolecular hydrogen bond to the nitrogen atom (N22) of the pyridine ring. The length of the hydrogen bond is 2.17 Å (Table 1) and forms an infinite chain parallel to the *c*-axis.

### Experimental

LDA (20 mmol) was added to dry THF (30 ml) in a three neck flask and cooled to 195 K. 4-Fluorophenylacetonitrile (14 mmol) in THF (10 ml) was added dropwise and the reaction was left stirring for 45 min. *N*-(2,4,6-trichlorophenyl)pyridine-4-carbohydrazonoyl chloride (5 mmol) was added slowly portionwise to the reaction. After about 1.0 h, the reaction was finished and left stirring to reach room temperature. Water (50 ml) was added to the reaction mixture and extracted with ethyl acetate (2x 50 mL). The organic layer was dried over Na_2_SO_4_. The organic layer was concentrated to about 5 ml and left overnight. The title compound was precipitated as a pale brown solid, filtered out, washed with petroleum ether. Yield 35%. The crystals for structure determination were obtained from recrystallization of the product from hot ethyl acetate.

### Refinement

Hydrogen atoms attached to carbons were placed at calculated positions with C—H = 0.95 Å (aromatic) or 0.98–0.99 Å (*sp*^3^ C-atom). Hydrogen atoms attached to nitrogen were located in diff. Fourier maps. All H atoms were refined in the riding-model approximation with isotropic displacement parameters (set at 1.2–1.5 times of the *U*_eq_ of the parent atom).

### Crystal data

**Table:**

C_20_H_12_Cl_3_FN_4_	*Z* = 2
*M**_r_* = 433.69	*F*(000) = 440
Triclinic, *P*1	*D*_x_ = 1.516 Mg m^−^^3^
Hall symbol: -P 1	Mo *K*α radiation, λ = 0.71073 Å
*a* = 10.2487 (5) Å	Cell parameters from 7293 reflections
*b* = 10.4643 (5) Å	θ = 2.2–27.7°
*c* = 10.5489 (5) Å	µ = 0.51 mm^−^^1^
α = 109.2377 (10)°	*T* = 173 K
β = 111.4008 (10)°	Plate, colourless
γ = 98.0304 (11)°	0.33 × 0.28 × 0.07 mm
*V* = 950.03 (8) Å^3^	

### Data collection

**Table:**

Bruker SMART APEXII diffractometer	4517 independent reflections
Radiation source: sealed Tube	3864 reflections with *I* > 2σ(*I*)
Graphite monochromator	*R*_int_ = 0.029
CCD scan	θ_max_ = 27.8°, θ_min_ = 2.2°
Absorption correction: multi-scan (*SADABS*; Bruker, 2006)	*h* = −13→13
*T*_min_ = 0.687, *T*_max_ = 0.746	*k* = −13→13
21201 measured reflections	*l* = −13→13

### Refinement

**Table:**

Refinement on *F*^2^	Primary atom site location: structure-invariant direct methods
Least-squares matrix: full	Secondary atom site location: difference Fourier map
*R*[*F*^2^ > 2σ(*F*^2^)] = 0.031	Hydrogen site location: inferred from neighbouring sites
*wR*(*F*^2^) = 0.079	H-atom parameters constrained
*S* = 1.03	*w* = 1/[σ^2^(*F*_o_^2^) + (0.0335*P*)^2^ + 0.4497*P*] where *P* = (*F*_o_^2^ + 2*F*_c_^2^)/3
4517 reflections	(Δ/σ)_max_ = 0.001
253 parameters	Δρ_max_ = 0.33 e Å^−^^3^
0 restraints	Δρ_min_ = −0.33 e Å^−^^3^

### Special details

**Table:**

Geometry. All e.s.d.'s (except the e.s.d. in the dihedral angle between two l.s. planes)are estimated using the full covariance matrix. The cell e.s.d.'s are takeninto account individually in the estimation of e.s.d.'s in distances, anglesand torsion angles; correlations between e.s.d.'s in cell parameters are onlyused when they are defined by crystal symmetry. An approximate (isotropic)treatment of cell e.s.d.'s is used for estimating e.s.d.'s involving l.s. planes.
Refinement. Refinement of *F*^2^ against ALL reflections. The weighted *R*-factor *wR* andgoodness of fit *S* are based on *F*^2^, conventional *R*-factors *R* are basedon *F*, with *F* set to zero for negative *F*^2^. The threshold expression of*F*^2^ > σ(*F*^2^) is used only for calculating *R*-factors(gt) *etc*. and isnot relevant to the choice of reflections for refinement. *R*-factors basedon *F*^2^ are statistically about twice as large as those based on *F*, and *R*-factors based on ALL data will be even larger.

### Fractional atomic coordinates and isotropic or equivalent isotropic displacement parameters (Å^2^)

**Table:**

	*x*	*y*	*z*	*U*_iso_*/*U*_eq_	
Cl1	1.11579 (4)	0.81835 (4)	0.49931 (4)	0.03113 (10)	
Cl2	0.55419 (4)	0.70476 (4)	0.42446 (4)	0.03333 (10)	
Cl3	0.97212 (5)	1.14538 (4)	0.90748 (5)	0.04454 (13)	
F1	0.50004 (11)	−0.13075 (9)	−0.22515 (12)	0.0397 (2)	
N1	0.79475 (14)	0.65783 (12)	0.33280 (12)	0.0233 (3)	
N2	0.78815 (13)	0.68969 (12)	0.21373 (12)	0.0218 (2)	
C3	0.75032 (15)	0.56326 (14)	0.10311 (14)	0.0195 (3)	
C4	0.73201 (16)	0.44990 (14)	0.14668 (15)	0.0210 (3)	
C5	0.76173 (16)	0.51568 (14)	0.29651 (15)	0.0225 (3)	
N6	0.76393 (17)	0.46090 (14)	0.39766 (14)	0.0333 (3)	
H6A	0.7576	0.5126	0.4816	0.050*	
H6B	0.7212	0.3720	0.3571	0.050*	
C7	0.83795 (16)	0.77123 (14)	0.47287 (14)	0.0212 (3)	
C8	0.98371 (16)	0.85674 (15)	0.55896 (15)	0.0220 (3)	
C9	1.02636 (16)	0.97336 (15)	0.69174 (15)	0.0241 (3)	
H9	1.1252	1.0322	0.7475	0.029*	
C10	0.92128 (17)	1.00187 (15)	0.74088 (16)	0.0260 (3)	
C11	0.77596 (17)	0.92009 (15)	0.66037 (16)	0.0258 (3)	
H11	0.7054	0.9417	0.6960	0.031*	
C12	0.73591 (16)	0.80573 (15)	0.52632 (15)	0.0228 (3)	
C13	0.67511 (15)	0.29689 (14)	0.05054 (15)	0.0203 (3)	
C14	0.73867 (17)	0.19851 (16)	0.09215 (16)	0.0261 (3)	
H14	0.8231	0.2304	0.1848	0.031*	
C15	0.67967 (18)	0.05394 (16)	−0.00078 (18)	0.0293 (3)	
H15	0.7226	−0.0129	0.0279	0.035*	
C16	0.55864 (17)	0.01080 (15)	−0.13404 (17)	0.0274 (3)	
C17	0.49321 (17)	0.10325 (16)	−0.18119 (16)	0.0272 (3)	
H17	0.4102	0.0701	−0.2752	0.033*	
C18	0.55209 (16)	0.24677 (15)	−0.08716 (16)	0.0235 (3)	
H18	0.5077	0.3123	−0.1172	0.028*	
C19	0.74226 (15)	0.55640 (14)	−0.04193 (14)	0.0192 (3)	
C20	0.67214 (16)	0.63729 (15)	−0.10978 (15)	0.0224 (3)	
H20	0.6215	0.6939	−0.0672	0.027*	
C21	0.67723 (17)	0.63399 (16)	−0.24026 (16)	0.0264 (3)	
H21	0.6265	0.6878	−0.2865	0.032*	
N22	0.74904 (15)	0.55985 (14)	−0.30536 (14)	0.0290 (3)	
C23	0.81503 (18)	0.48164 (17)	−0.23960 (17)	0.0292 (3)	
H23	0.8670	0.4280	−0.2832	0.035*	
C24	0.81216 (16)	0.47424 (15)	−0.11190 (16)	0.0242 (3)	
H24	0.8574	0.4138	−0.0725	0.029*	

### Atomic displacement parameters (Å^2^)

**Table:**

	*U*^11^	*U*^22^	*U*^33^	*U*^12^	*U*^13^	*U*^23^
Cl1	0.03306 (19)	0.0364 (2)	0.02770 (18)	0.01592 (16)	0.01643 (15)	0.01189 (16)
Cl2	0.02807 (19)	0.0373 (2)	0.02116 (17)	−0.00504 (15)	0.00680 (14)	0.00688 (15)
Cl3	0.0481 (2)	0.0315 (2)	0.0318 (2)	−0.00855 (18)	0.02382 (19)	−0.01149 (17)
F1	0.0488 (6)	0.0173 (4)	0.0460 (6)	0.0034 (4)	0.0257 (5)	0.0021 (4)
N1	0.0368 (7)	0.0182 (6)	0.0127 (5)	0.0038 (5)	0.0108 (5)	0.0058 (4)
N2	0.0309 (6)	0.0211 (6)	0.0135 (5)	0.0046 (5)	0.0100 (5)	0.0083 (4)
C3	0.0231 (6)	0.0203 (6)	0.0141 (6)	0.0052 (5)	0.0082 (5)	0.0063 (5)
C4	0.0282 (7)	0.0196 (6)	0.0153 (6)	0.0060 (5)	0.0100 (5)	0.0073 (5)
C5	0.0320 (7)	0.0190 (6)	0.0163 (6)	0.0062 (6)	0.0109 (6)	0.0075 (5)
N6	0.0631 (9)	0.0211 (6)	0.0191 (6)	0.0100 (6)	0.0213 (6)	0.0094 (5)
C7	0.0319 (7)	0.0170 (6)	0.0127 (6)	0.0046 (5)	0.0088 (5)	0.0061 (5)
C8	0.0280 (7)	0.0227 (7)	0.0180 (6)	0.0088 (6)	0.0109 (5)	0.0103 (5)
C9	0.0259 (7)	0.0221 (7)	0.0189 (6)	0.0022 (6)	0.0077 (6)	0.0066 (5)
C10	0.0341 (8)	0.0197 (7)	0.0177 (6)	0.0015 (6)	0.0122 (6)	0.0020 (5)
C11	0.0313 (8)	0.0238 (7)	0.0222 (7)	0.0034 (6)	0.0158 (6)	0.0066 (6)
C12	0.0257 (7)	0.0219 (7)	0.0166 (6)	0.0000 (5)	0.0076 (5)	0.0078 (5)
C13	0.0280 (7)	0.0187 (6)	0.0176 (6)	0.0059 (5)	0.0139 (5)	0.0072 (5)
C14	0.0336 (8)	0.0258 (7)	0.0214 (7)	0.0107 (6)	0.0127 (6)	0.0111 (6)
C15	0.0410 (9)	0.0241 (7)	0.0341 (8)	0.0150 (7)	0.0227 (7)	0.0159 (6)
C16	0.0365 (8)	0.0164 (6)	0.0309 (8)	0.0038 (6)	0.0228 (7)	0.0045 (6)
C17	0.0292 (7)	0.0242 (7)	0.0224 (7)	0.0029 (6)	0.0113 (6)	0.0047 (6)
C18	0.0281 (7)	0.0208 (7)	0.0223 (7)	0.0073 (6)	0.0121 (6)	0.0088 (5)
C19	0.0217 (6)	0.0185 (6)	0.0135 (6)	0.0004 (5)	0.0071 (5)	0.0050 (5)
C20	0.0290 (7)	0.0210 (7)	0.0189 (6)	0.0070 (6)	0.0128 (6)	0.0079 (5)
C21	0.0360 (8)	0.0259 (7)	0.0194 (7)	0.0083 (6)	0.0128 (6)	0.0116 (6)
N22	0.0401 (7)	0.0291 (7)	0.0199 (6)	0.0067 (6)	0.0169 (5)	0.0098 (5)
C23	0.0367 (8)	0.0301 (8)	0.0247 (7)	0.0111 (6)	0.0194 (6)	0.0086 (6)
C24	0.0285 (7)	0.0251 (7)	0.0210 (7)	0.0091 (6)	0.0119 (6)	0.0102 (6)

### Geometric parameters (Å, º)

**Table:**

Cl1—C8	1.7310 (15)	C11—H11	0.9500
Cl2—C12	1.7266 (15)	C13—C18	1.3969 (19)
Cl3—C10	1.7289 (14)	C13—C14	1.398 (2)
F1—C16	1.3677 (16)	C14—C15	1.396 (2)
N1—C5	1.3649 (18)	C14—H14	0.9500
N1—N2	1.3833 (16)	C15—C16	1.368 (2)
N1—C7	1.4170 (16)	C15—H15	0.9500
N2—C3	1.3310 (17)	C16—C17	1.374 (2)
C3—C4	1.4190 (19)	C17—C18	1.390 (2)
C3—C19	1.4788 (18)	C17—H17	0.9500
C4—C5	1.3921 (18)	C18—H18	0.9500
C4—C13	1.4730 (18)	C19—C24	1.3918 (19)
C5—N6	1.3627 (18)	C19—C20	1.3925 (19)
N6—H6A	0.9044	C20—C21	1.3857 (19)
N6—H6B	0.8528	C20—H20	0.9500
C7—C12	1.394 (2)	C21—N22	1.339 (2)
C7—C8	1.399 (2)	C21—H21	0.9500
C8—C9	1.3856 (19)	N22—C23	1.340 (2)
C9—C10	1.383 (2)	C23—C24	1.385 (2)
C9—H9	0.9500	C23—H23	0.9500
C10—C11	1.383 (2)	C24—H24	0.9500
C11—C12	1.3872 (19)		
			
C5—N1—N2	112.70 (11)	C18—C13—C14	118.19 (13)
C5—N1—C7	128.89 (12)	C18—C13—C4	119.28 (12)
N2—N1—C7	118.37 (11)	C14—C13—C4	122.54 (13)
C3—N2—N1	103.52 (11)	C15—C14—C13	120.83 (14)
N2—C3—C4	112.95 (12)	C15—C14—H14	119.6
N2—C3—C19	118.76 (12)	C13—C14—H14	119.6
C4—C3—C19	128.15 (12)	C16—C15—C14	118.46 (14)
C5—C4—C3	104.41 (12)	C16—C15—H15	120.8
C5—C4—C13	127.41 (13)	C14—C15—H15	120.8
C3—C4—C13	127.71 (12)	F1—C16—C15	118.68 (14)
N6—C5—N1	122.51 (12)	F1—C16—C17	118.28 (14)
N6—C5—C4	131.05 (13)	C15—C16—C17	123.04 (13)
N1—C5—C4	106.42 (12)	C16—C17—C18	117.99 (14)
C5—N6—H6A	120.3	C16—C17—H17	121.0
C5—N6—H6B	113.1	C18—C17—H17	121.0
H6A—N6—H6B	117.0	C17—C18—C13	121.49 (13)
C12—C7—C8	117.70 (12)	C17—C18—H18	119.3
C12—C7—N1	121.29 (13)	C13—C18—H18	119.3
C8—C7—N1	120.93 (13)	C24—C19—C20	117.29 (12)
C9—C8—C7	121.78 (13)	C24—C19—C3	120.87 (12)
C9—C8—Cl1	118.35 (11)	C20—C19—C3	121.74 (12)
C7—C8—Cl1	119.86 (10)	C21—C20—C19	119.06 (13)
C10—C9—C8	118.23 (13)	C21—C20—H20	120.5
C10—C9—H9	120.9	C19—C20—H20	120.5
C8—C9—H9	120.9	N22—C21—C20	124.20 (14)
C9—C10—C11	122.19 (13)	N22—C21—H21	117.9
C9—C10—Cl3	119.16 (11)	C20—C21—H21	117.9
C11—C10—Cl3	118.65 (12)	C21—N22—C23	116.10 (13)
C10—C11—C12	118.28 (14)	N22—C23—C24	124.00 (14)
C10—C11—H11	120.9	N22—C23—H23	118.0
C12—C11—H11	120.9	C24—C23—H23	118.0
C11—C12—C7	121.79 (13)	C23—C24—C19	119.25 (14)
C11—C12—Cl2	118.38 (12)	C23—C24—H24	120.4
C7—C12—Cl2	119.83 (11)	C19—C24—H24	120.4
			
C5—N1—N2—C3	−0.07 (16)	C10—C11—C12—Cl2	−179.22 (12)
C7—N1—N2—C3	177.83 (12)	C8—C7—C12—C11	−0.3 (2)
N1—N2—C3—C4	0.03 (16)	N1—C7—C12—C11	−177.31 (13)
N1—N2—C3—C19	−175.96 (12)	C8—C7—C12—Cl2	179.62 (11)
N2—C3—C4—C5	0.02 (17)	N1—C7—C12—Cl2	2.64 (19)
C19—C3—C4—C5	175.55 (13)	C5—C4—C13—C18	127.95 (16)
N2—C3—C4—C13	172.59 (13)	C3—C4—C13—C18	−43.0 (2)
C19—C3—C4—C13	−11.9 (2)	C5—C4—C13—C14	−51.4 (2)
N2—N1—C5—N6	178.89 (14)	C3—C4—C13—C14	137.65 (16)
C7—N1—C5—N6	1.3 (2)	C18—C13—C14—C15	−0.7 (2)
N2—N1—C5—C4	0.09 (17)	C4—C13—C14—C15	178.67 (14)
C7—N1—C5—C4	−177.54 (14)	C13—C14—C15—C16	0.4 (2)
C3—C4—C5—N6	−178.73 (16)	C14—C15—C16—F1	−179.70 (13)
C13—C4—C5—N6	8.7 (3)	C14—C15—C16—C17	0.6 (2)
C3—C4—C5—N1	−0.07 (16)	F1—C16—C17—C18	179.13 (13)
C13—C4—C5—N1	−172.66 (14)	C15—C16—C17—C18	−1.1 (2)
C5—N1—C7—C12	−77.8 (2)	C16—C17—C18—C13	0.8 (2)
N2—N1—C7—C12	104.65 (16)	C14—C13—C18—C17	0.1 (2)
C5—N1—C7—C8	105.30 (18)	C4—C13—C18—C17	−179.29 (13)
N2—N1—C7—C8	−72.23 (18)	N2—C3—C19—C24	130.10 (15)
C12—C7—C8—C9	−1.0 (2)	C4—C3—C19—C24	−45.2 (2)
N1—C7—C8—C9	175.96 (13)	N2—C3—C19—C20	−46.22 (19)
C12—C7—C8—Cl1	179.43 (11)	C4—C3—C19—C20	138.48 (15)
N1—C7—C8—Cl1	−3.59 (18)	C24—C19—C20—C21	−1.0 (2)
C7—C8—C9—C10	1.9 (2)	C3—C19—C20—C21	175.42 (13)
Cl1—C8—C9—C10	−178.53 (11)	C19—C20—C21—N22	−1.7 (2)
C8—C9—C10—C11	−1.5 (2)	C20—C21—N22—C23	2.2 (2)
C8—C9—C10—Cl3	179.15 (11)	C21—N22—C23—C24	0.1 (2)
C9—C10—C11—C12	0.2 (2)	N22—C23—C24—C19	−2.7 (2)
Cl3—C10—C11—C12	179.56 (11)	C20—C19—C24—C23	3.1 (2)
C10—C11—C12—C7	0.7 (2)	C3—C19—C24—C23	−173.41 (13)

### Hydrogen-bond geometry (Å, º)

**Table:**

*D*—H···*A*	*D*—H	H···*A*	*D*···*A*	*D*—H···*A*
N6—H6*A*···N22^i^	0.90	2.17	3.0275 (17)	157

Symmetry code: (i) *x*, *y*, *z*+1.
